# Supplementary material for: Female sex is a risk factor for exacerbated lipid peroxidation and disease in murine retinitis pigmentosa
Source: Redox Biol. 2025 Dec 23;90:103987. doi: 10.1016/j.redox.2025.103987 (PMC12870477; doi:10.1016/j.redox.2025.103987)
Supplement: Supporting_information [file mmc4.docx]

Supporting Information

**Female sex is a risk factor for exacerbated lipid peroxidation and disease in murine retinitis pigmentosa**

Katri Vainionpää ^1^, Anna Kalatanova ^1^, Umair Seemab ^1^, Ahmed B Montaser ^1^, Henri Leinonen ^1^*.

^1^ School of Pharmacy, Faculty of Health Sciences, University of Eastern Finland, Kuopio, Finland.

*Correspondence: Henri Leinonen, University of Eastern Finland, School of Pharmacy, Yliopistonranta 1 C, Kuopio 70211, Finland, tel. +358 50 430 3196, email: [henri.leinonen@uef.fi](mailto:henri.leinonen@uef.fi)

**This file includes:**

Figures S1 to S15

Tables S1 to S4

Legends for Supplemental Data 1 to 3

SI References


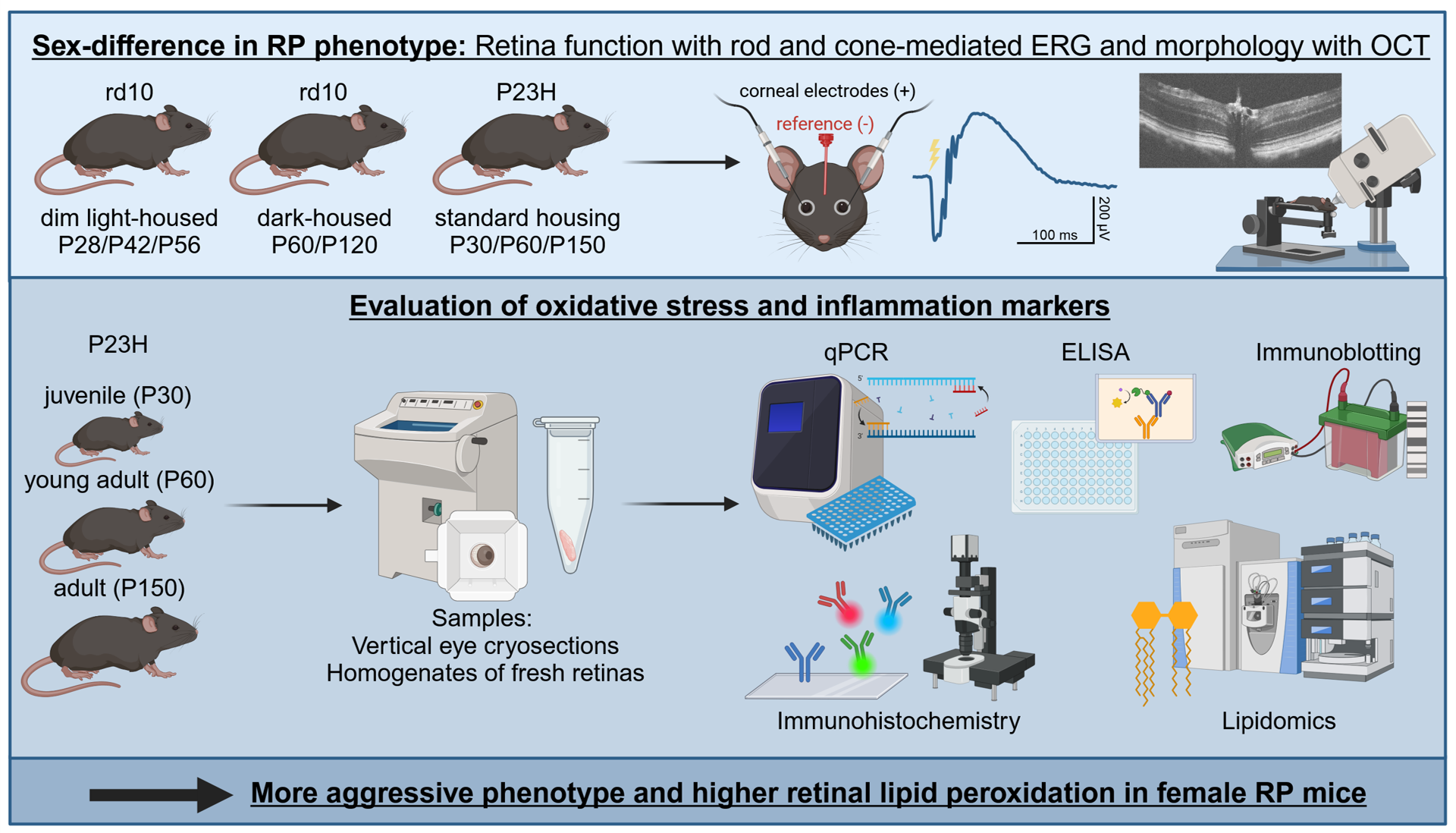


**Figure S1. Graphical abstract of the study.** Summary of used animal paradigms and methods.

**
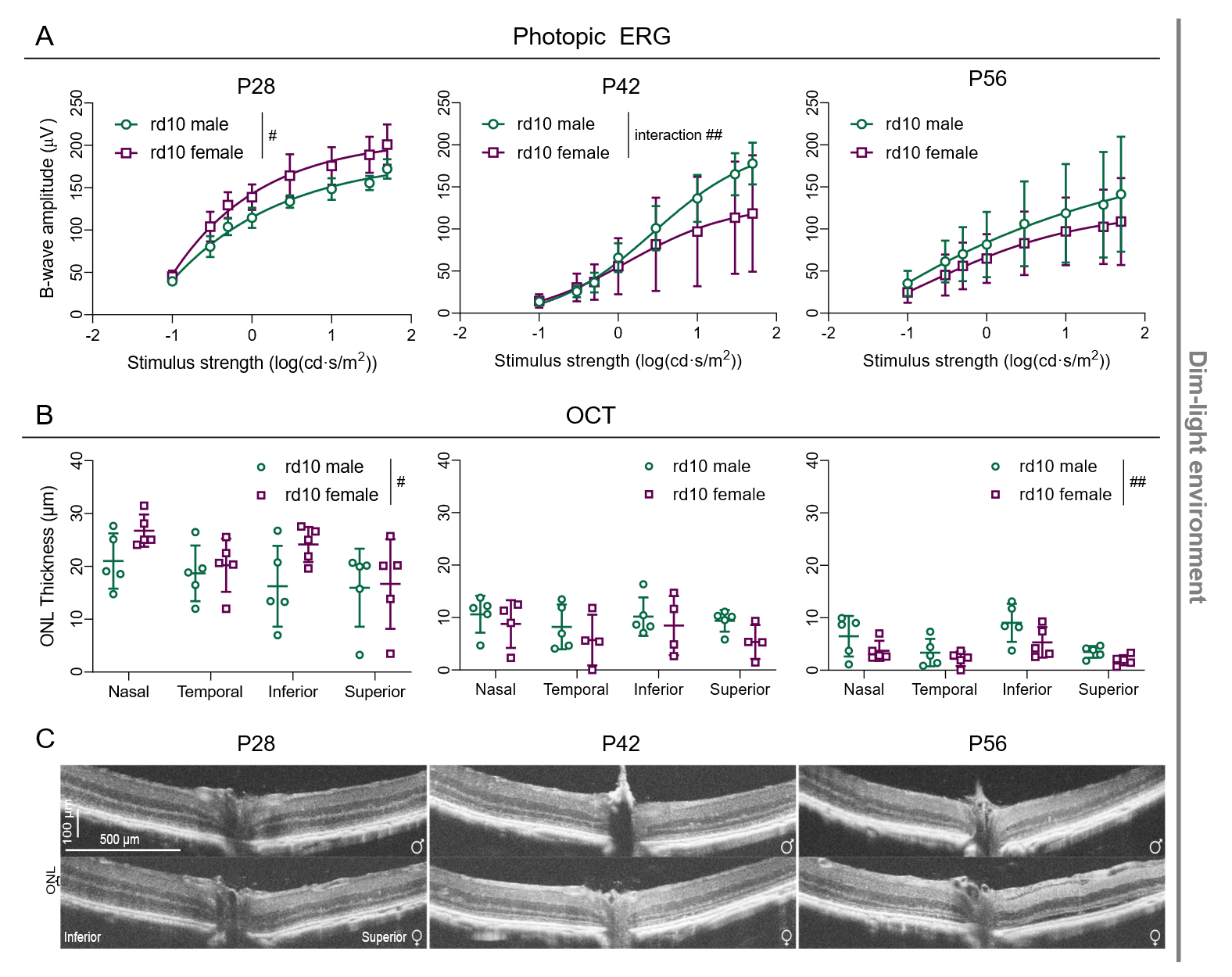
**

**Figure S2. Disease phenotype worsening in dim-light reared (< 1 lux) rd10 female mice coincides with sexual maturity.** A) Photopic electroretinogram (ERG) b-wave (blue stimulus), mean ± standard deviation (SD). P28, n = 5 males (m), n = 5 females (f); P42, n = 5 m, n = 4 f; P56, n = 5 m, n = 5 f. B) Outer nuclear layer (ONL) thicknesses at each timepoint, mean ± SD. C) Representative vertically oriented optical coherence tomography (OCT) images from each timepoint from male and female rd10 mice. The hashtags indicate significant between-subjects two-way ANOVA main effects (repeated measures (RM) ANOVA used in panel A and regular ANOVA in panel B): # = p < 0.05 and ## = p < 0.01.


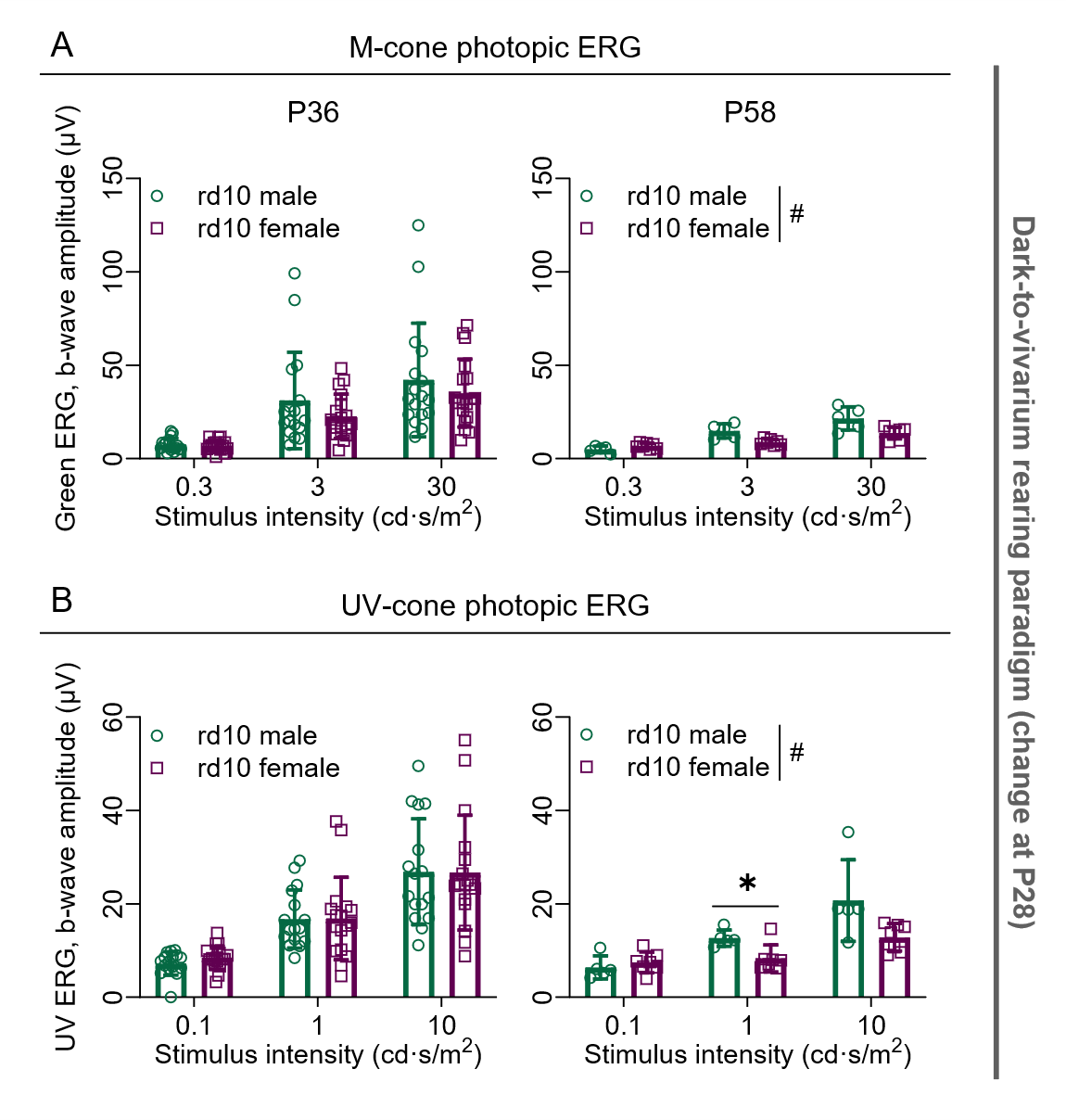


**Figure S3. Reanalysis of publicly available source data from Leinonen *et al.* [1] reveals greater loss of photopic ERG amplitudes in rd10 females as mice reach adulthood and sexual maturity.** Mean b-wave amplitudes from ERGs elicited with A) green (M-cone-dominant assay) and B) UV light flashes (UV-cone-dominant assay). Mice were dark-reared until P28 and then moved to regular vivarium conditions. A subset of mice was recorded at P36 (left panels) and P58 (right panels). Hashtags and asterisks indicate significant two-way ANOVA main effects and Šídák's posthoc test results, respectively: #/* = p < 0.05.


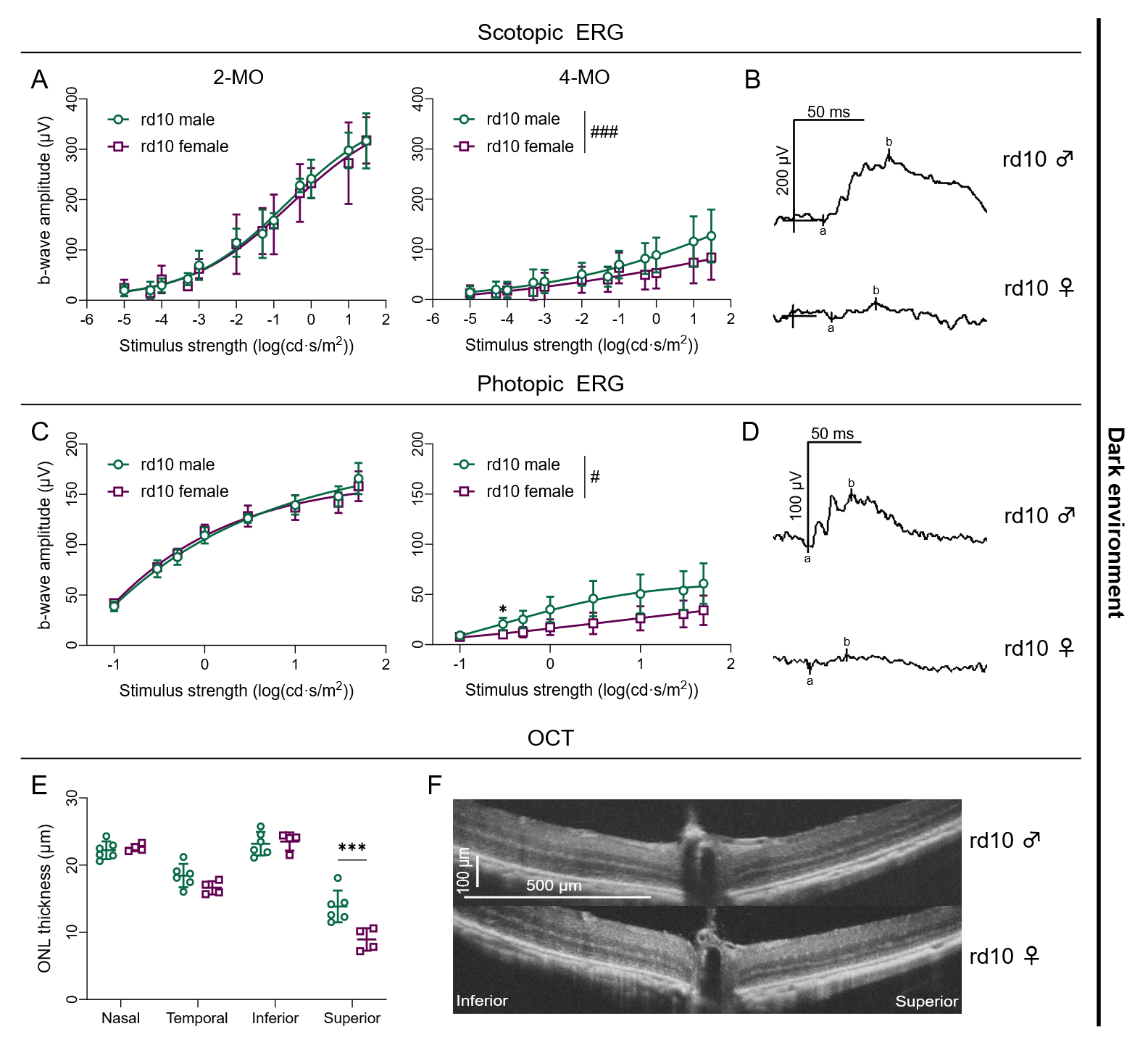


**Figure S4. Sex difference in adult rd10 mice is clear when constantly reared in full darkness.** This supplementary figure extends the data presented in main Figure 1. A) Scotopic ERG b-wave, mean ± SD. 2-month-old (MO), n = 6 m, n = 4 f; 4-MO, n = 15 m, n = 13 f. B) Representative scotopic ERG waveforms in 4-month-old mice (stimulus intensity: 1 cd • s/m2). C) Photopic ERG b-wave (blue stimulus), mean ± SD. 2-MO, n = 6 m + 4 f; 4-MO n = 7 m + 6 f. D) Representative photopic ERG waveforms in 4-month-old mice (stimulus intensity: 10 cd • s/m2). E) ONL thicknesses in 2-month-old mice, mean ± SD. F) Representative vertical OCT images from 2-month-old rd10 male and female mice. The hashtags and asterisks indicate significant between-subjects two-way ANOVA main effects (RM ANOVA used in panels A and C, and regular ANOVA in panel E) and Šídák's posthoc test results, respectively: #/* = p < 0.05, ##/** = p < 0.01 and ###/*** = p < 0.001.


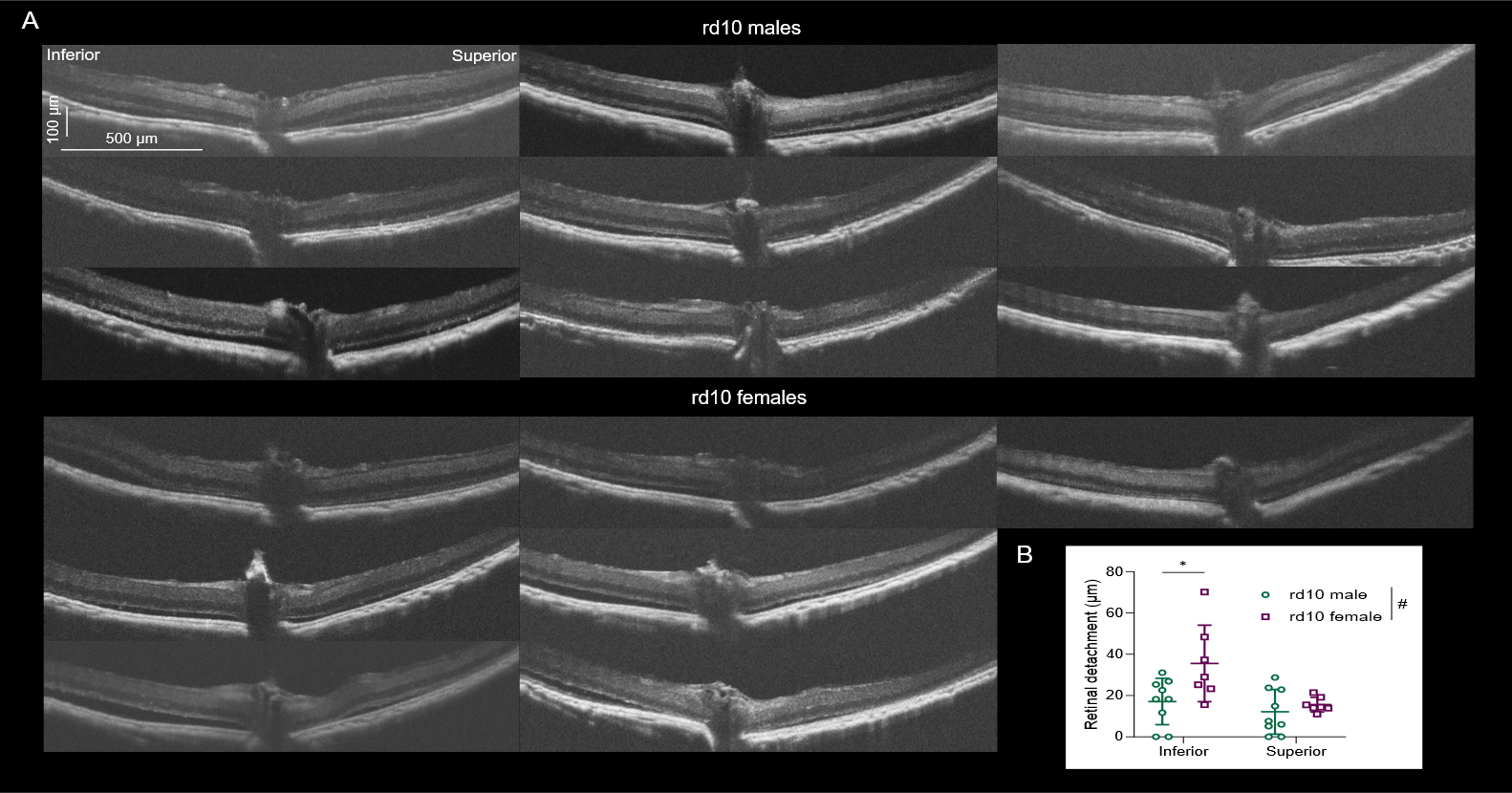


**Figure S5. Retinal detachment in the vertical retina is more severe in 4-month-old rd10 female mice.** This supplementary figure extends the data presented in main Figure 1. A) Vertically oriented example OCT images. B) Retinal detachment as measured from the highest point of detachment in inferior and superior orientations. The hashtags and asterisks indicate significant between-subjects ANOVA main effects and posthoc test results, respectively: #/* = p < 0.05, mean ± SD.


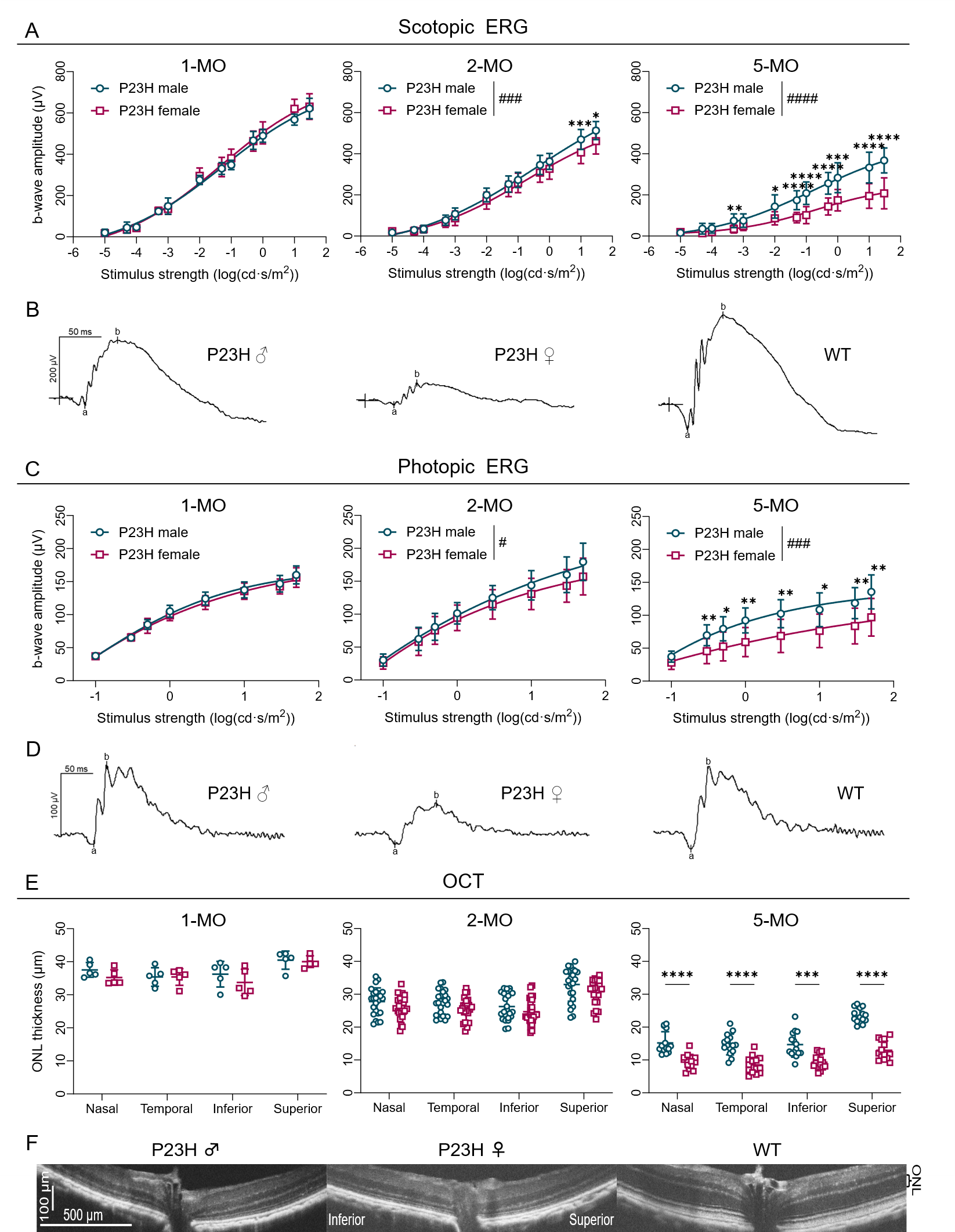


**Figure S6. Disease phenotype is accelerated in P23H female mice compared to males after puberty.** This supplementary figure extends the data presented in main Figure 1. A) Scotopic b-wave, mean ± SD. 1-MO, n = 5 m + 5 f; 2-MO, n = 24 m, n = 26 f; 5-MO, n = 16 m, n = 14 f. B) Representative scotopic ERG waveforms in 5-month-old mice (stimulus intensity: 0.05 cd • s/m2). C) Photopic ERG b-wave (blue stimulus), mean ± SD. 1-MO, n = 5 m, n = 5 f; 2-MO, n = 24 m, n = 26 f; 5-MO, n = 15 m, n = 14 f. D) Representative photopic ERG waveforms in 5-month-old mice (stimulus intensity: 10 cd • s/m2). E) ONL thicknesses, mean ± SD. F) Representative vertical OCT images from 5-month-old male and female P23H, and WT mice. The hashtags and asterisks indicate significant between-subjects two-way ANOVA main effects (RM ANOVA used in panels A and C, and regular ANOVA in panel E) and Šídák's posthoc test results, respectively: #/* = p < 0.05, ##/** = p < 0.01, ###/*** = p < 0.001 and ####/**** = p < 0.0001.


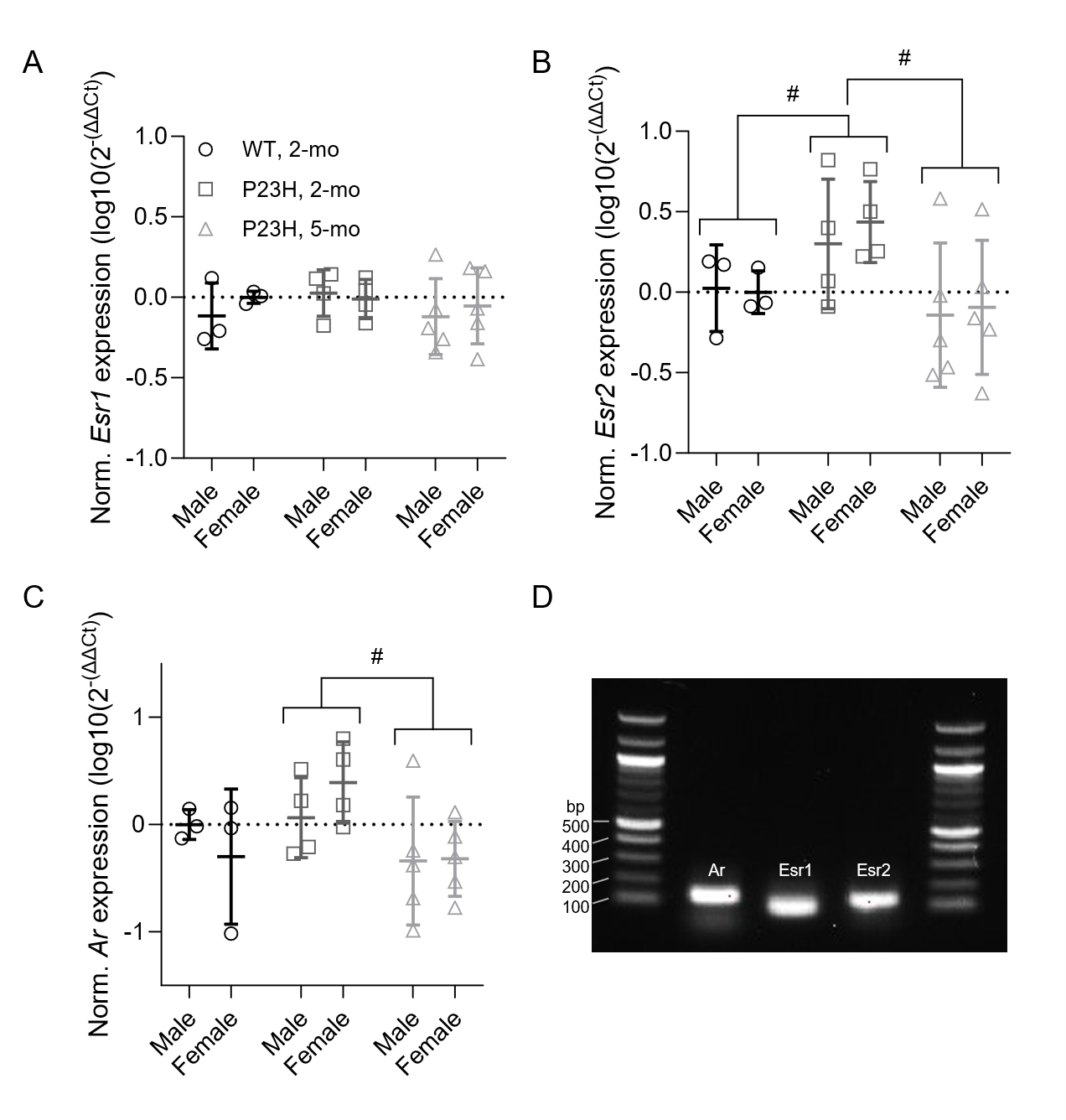


**Figure S7. No sex differences in the expression of estrogen receptor 1 (*Esr1*) and 2 (*Esr2*) , and androgen receptor (*Ar*) genes.** The relative gene expression of A) *Esr1*, B) *Esr2*, and C) *Ar* in 2-month-old WT and P23H, and 5-month-old P23H retinas as measured with real-time quantitative PCR, log10(2^-(ΔΔCt)^). The hashtags indicate significant main two-way ANOVA effect, # < 0.05. Data is presented as mean ± SD. D) Primer specificity was tested with electrophoresis.


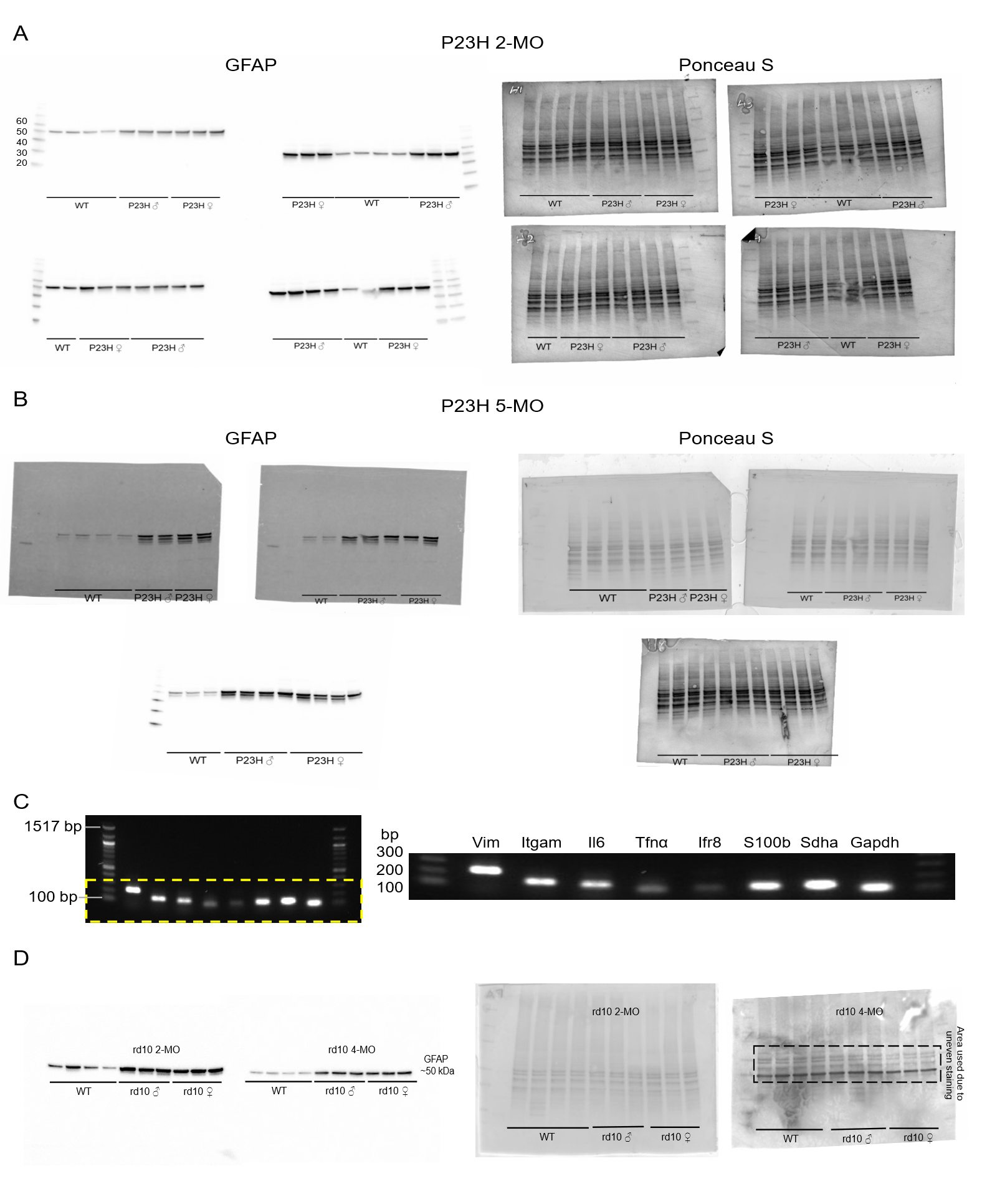
**Figure S8. Full immunoblots (A,B), and endpoint PCR with primers used in qPCR.** This figure relates to main text Figures 2 (A-C) and 4 (D). All analysed glial fibrillary acidic protein (GFAP) and Ponceau S stained immunoblots with A) 2-month-old and B) 5-month-old P23H and WT retina samples. C) Electrophoresis with example sample after amplification with vimentin (*Vim*), integrin subunit alpha M (*Itgam*), interleukin 6 (*Il6*), tumor necrosis factor alpha (*Tnfa*), interferon regulatory factor 8 (*Irf8*), S100 calcium-binding protein B (*S100b*), succinate dehydrogenase complex flavoprotein subunit A (*Sdha*) and glyceraldehyde 3-phosphate dehydrogenase (*Gapdh*) primers. D) All analysed GFAP and Ponceau S stained immunoblots with rd10 and WT retina samples.


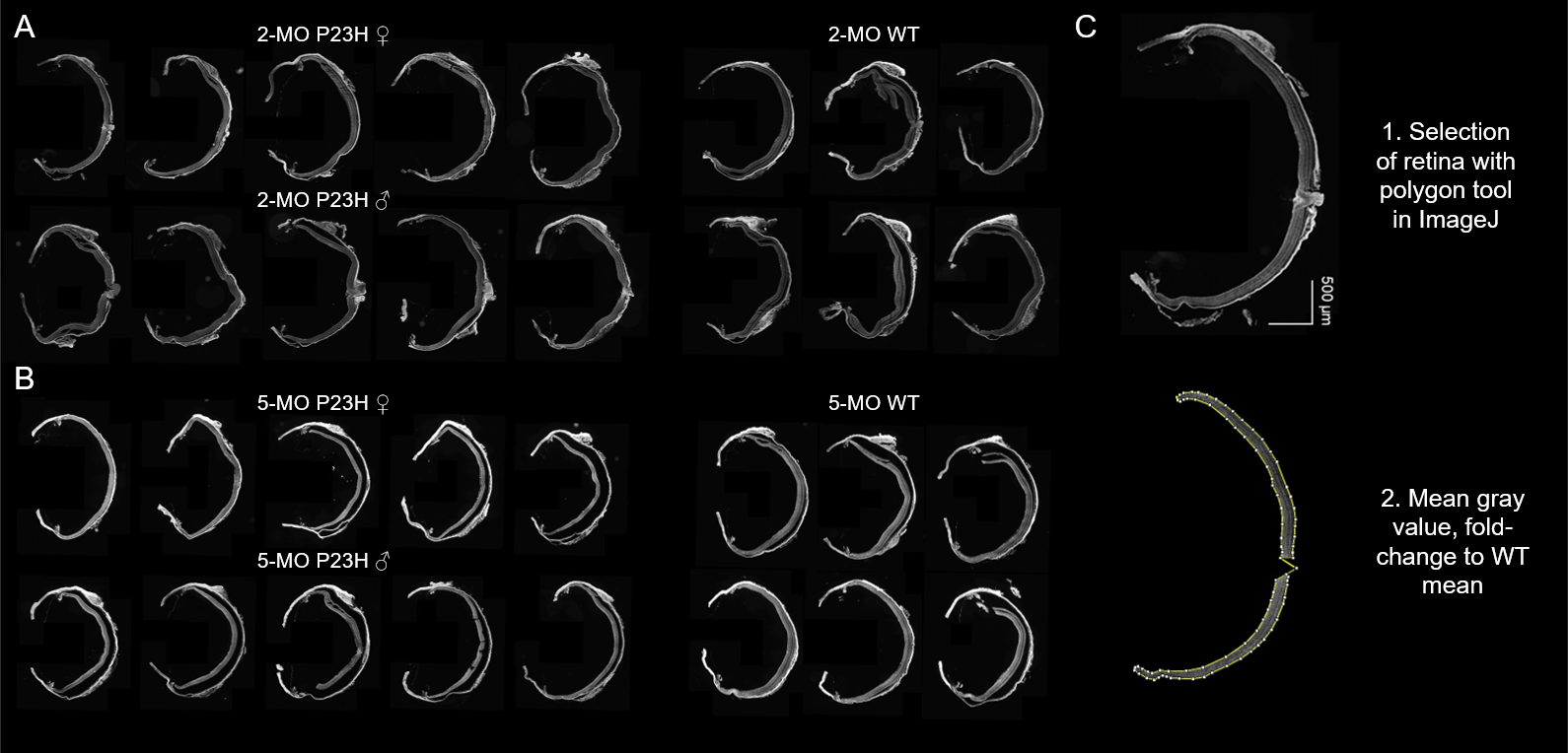
 **Figure S9. 4-HNE signal from all samples used for analysis in main text Figure 3B.** 4-HNE-stained cryosections from vertically oriented eye cups from A) 2- and B) 5-month-old P23H and WT mice. C) Example of retinal area selection for quantification.


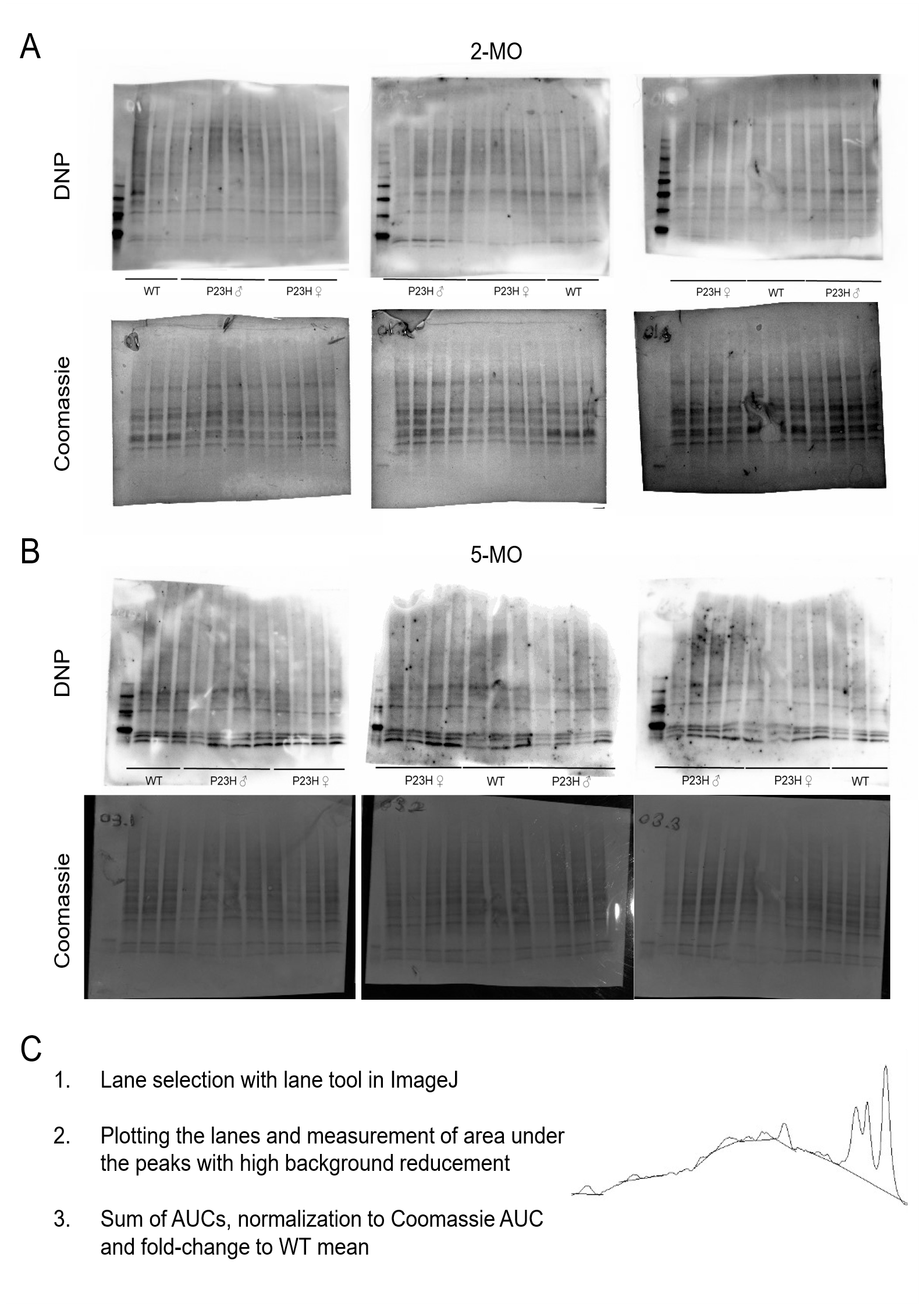
**Figure S10. Measurement of protein carbonylation by immunoblotting.** Relates to main text Figure 3E. All analysed 2,4-dinitrophenylhydrazine (DNP) and Coomassie stained blots with A) 2- and B) 5-month-old P23H and WT retina samples. C) Workflow for image analysis.


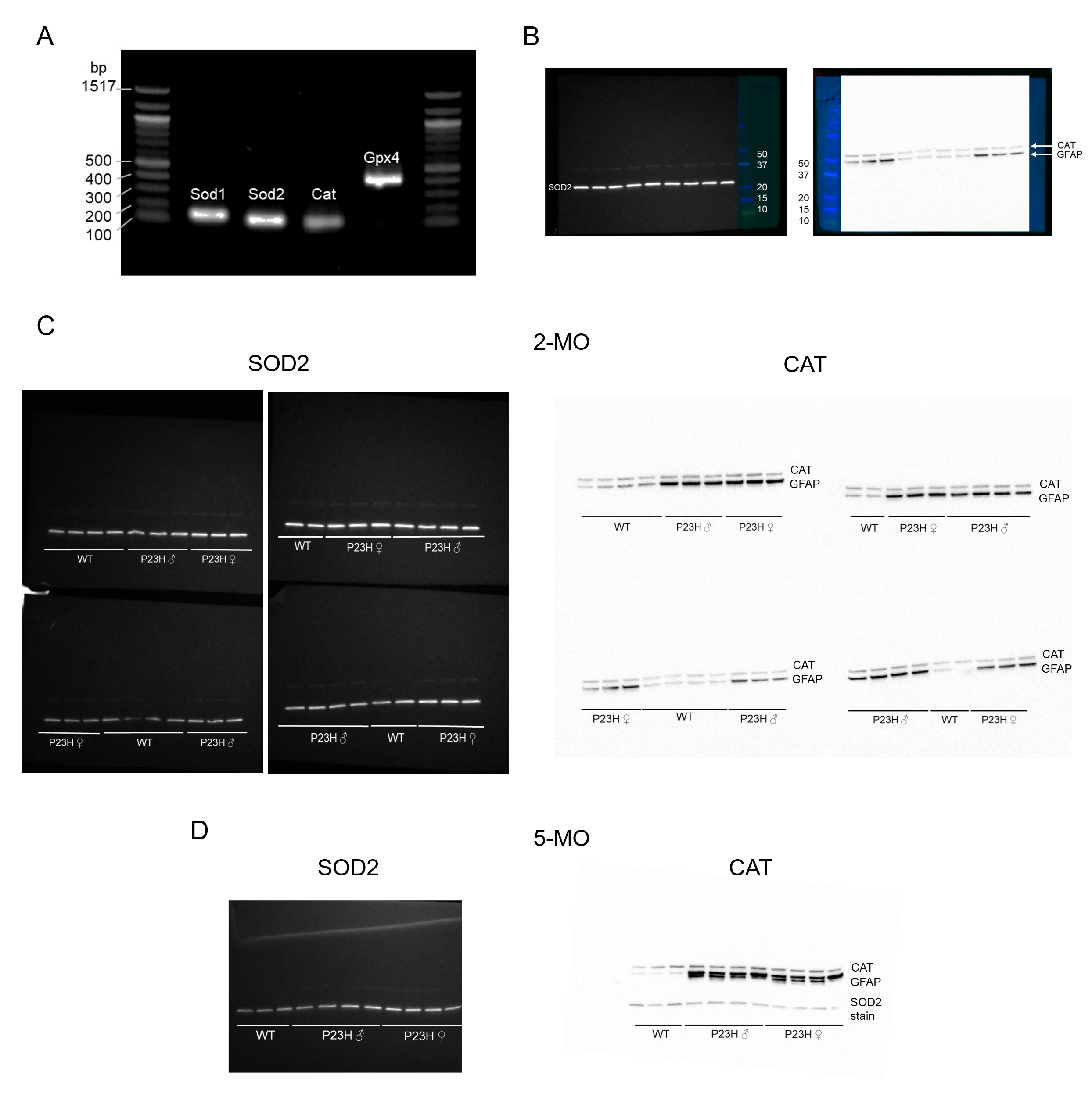


**Figure S11. Assessment of antioxidative system in male and female P23H retinas.** A) Electrophoresis with example samples after amplification with superoxide dismutase 1 (*Sod1)*, superoxide dismutase 2 (*Sod2*), catalase (*Cat*) and glutathione peroxidase 4 (*Gpx4*) primers. Relates to main text Figures 3G-J. B) Merge images of membranes with SOD2 and CAT (and glial fibrillary acidic protein (GFAP) remnant) signals with molecular weight markers. Full images of immunoblots stained with SOD2 and CAT with retina samples from C) 2- and D) 5-month-old P23H and WT mice. Panels B-D relate to main text Figures 3K-M.

**
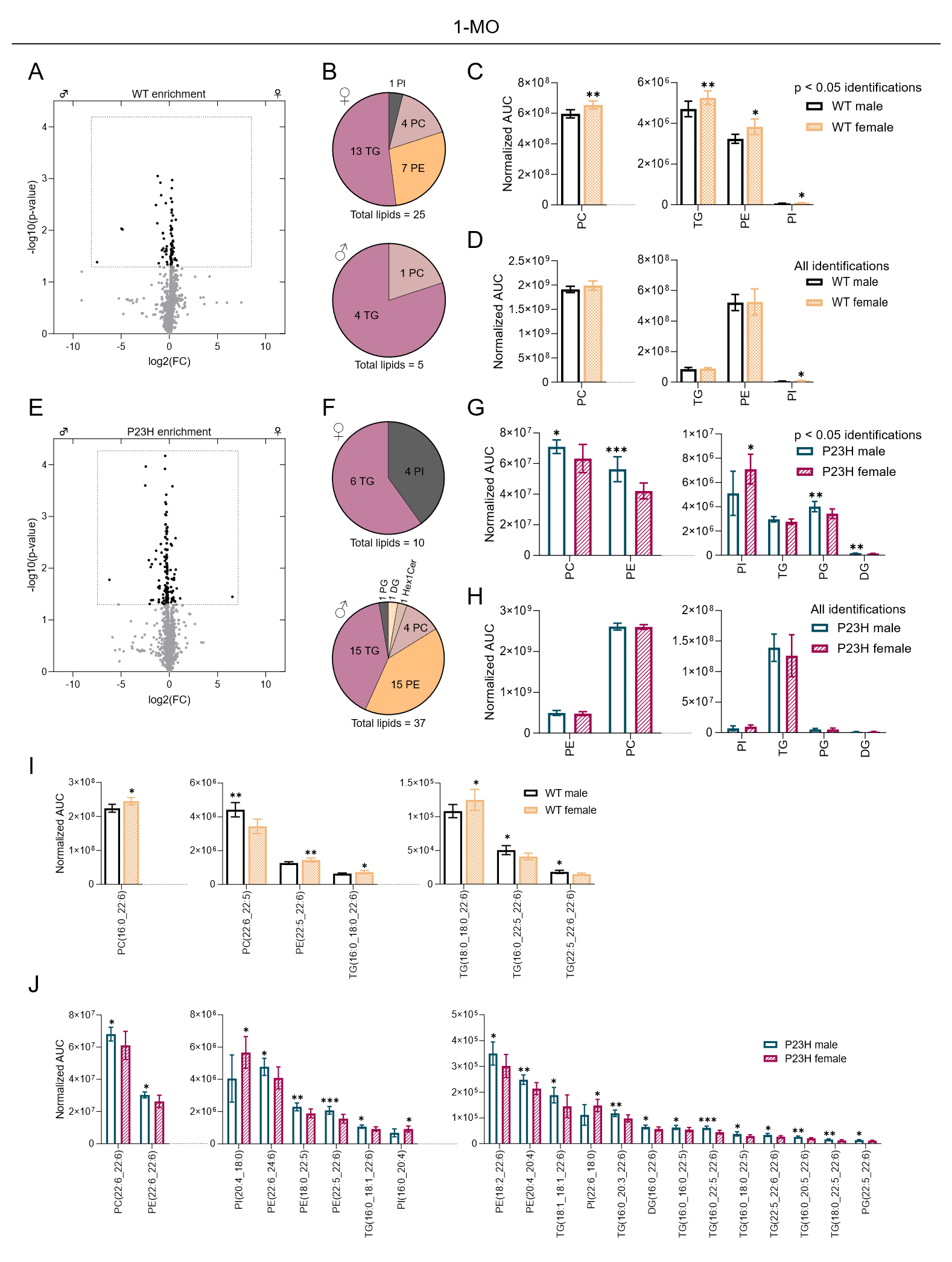
**

**Figure S12. Comparison of retinal lipids between 1-month-old WT male and WT female, and P23H male and P23H female mice.** A) Volcano plot of all identified lipids in WT mice. Differentially expressed lipids (DELs, p < 0.05) between WT males (n = 7) and females (n = 5) are shown in black. B) Number of enriched DELs per lipid class in WT females and males. C) Lipid group expression comparison between WT male and female mice based on DELs only. D) Lipid group expression comparison between WT male and female mice based on all lipid identifications. E) Volcano plot of all identified lipids in P23H mice (n = 9 males; n = 8 females). F) Number of enriched DELs per lipid class in P23H females and males. G) Lipid group expression comparison between P23H male and female mice based on DELs only. H) Lipid group expression comparison between P23H male and female mice based on all lipid identifications. I) DELs with 20:4/22:5/22:6 carbon/double-bond sidechains between WT male and female mice, and J) between P23H male and female mice. In all graphs, the asterisks indicate significant t-test results, * = p < 0.05, ** = p < 0.01 and *** = p < 0.001. The bar graph data is presented as mean ± SD. DG = diglyceride, PC = phosphatidylcholine, PE = phosphatidylethanolamine, PI = phosphatidylinositol, PG = phosphatidylglycerol, TG = triglyceride.

**
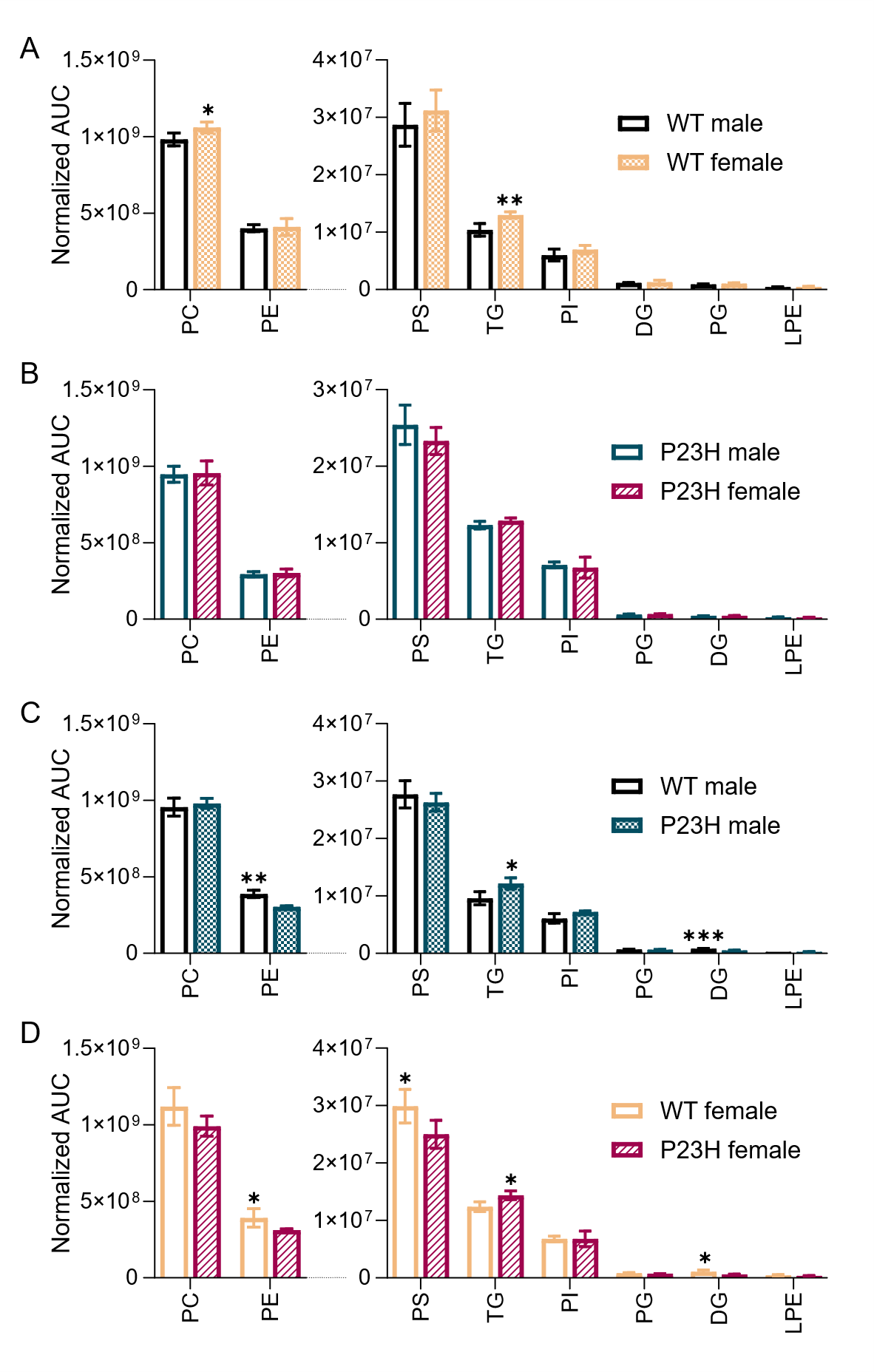
**

**Figure S13. Lipid group expression comparisons with all lipid identification with LC-PUFA (20:4, 22:5 or 22:6) sidechains, 2-month-old WT and P23H retinas.** Comparisons are between A) WT male (n = 4) and WT (n = 4) female, B) P23H male (n = 3) and P23H female (n = 4), C) WT male and P23H male, and D) WT female and P23H female retinas, mean ±SD. In all graphs, the asterisks indicate significant t-test results, * = p < 0.05, ** = p < 0.01 and *** = p < 0.001. DG = diglyceride, LPE = lysophosphatidylethanolamine, PC = phosphatidylcholine, PE = phosphatidylethanolamine, PG = phosphatidylglycerol, PI = phosphatidylinositol, PS = phosphatidylserine, TG = triglyceride.


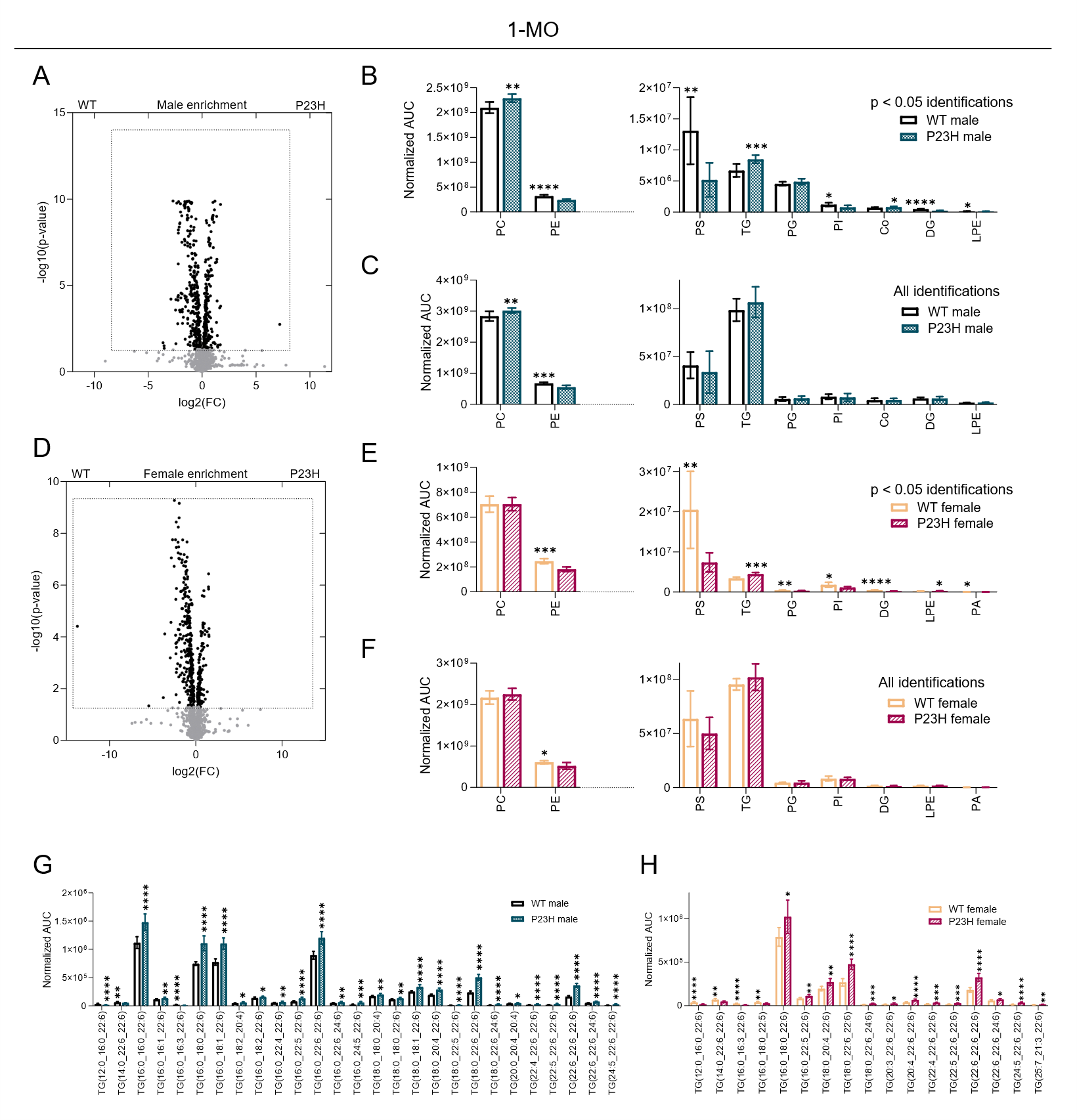


**Figure S14. Analysis of differentially expressed lipids between genotypes for both sexes at the age of 1 month.** A) Volcano plot of all identified lipids in WT and P23H males. Differentially expressed lipids (DELs) between WT males (n = 7) and P23H males (n = 9) are shown in black. B) Lipid group expression comparison between WT males and P23H males based on DELs only. C) Lipid group expression comparison between WT males and P23H males based on all lipid identifications. D) Volcano plot of all identified lipids in WT and P23H females. DELs between WT females (n = 5) and P23H females (n = 8) are shown in black. E) Lipid group expression comparison between WT females and P23H females based on DELs only. F) Lipid group expression comparison between WT females and P23H females based on all lipid identifications. G) Differentially expressed TGs with 20:4/22:5/22:6 carbon/double-bond sidechains between WT males and P23H males, and H) between WT females and P23H females. In all graphs, the asterisks indicate significant t-test results, * = p < 0.05, ** = p < 0.01, *** = p < 0.001 and **** = p < 0.0001. The bar graph data is presented as mean ±SD. Co = coenzyme Q, DG = diglyceride, LPE = lysophosphatidylethanolamine, PA = phosphatidic acid, PC = phosphatidylcholine, PE = phosphatidylethanolamine, PG = phosphatidylglycerol, PI = phosphatidylinositol, PS = phosphatidylserine, TG = triglyceride.

**
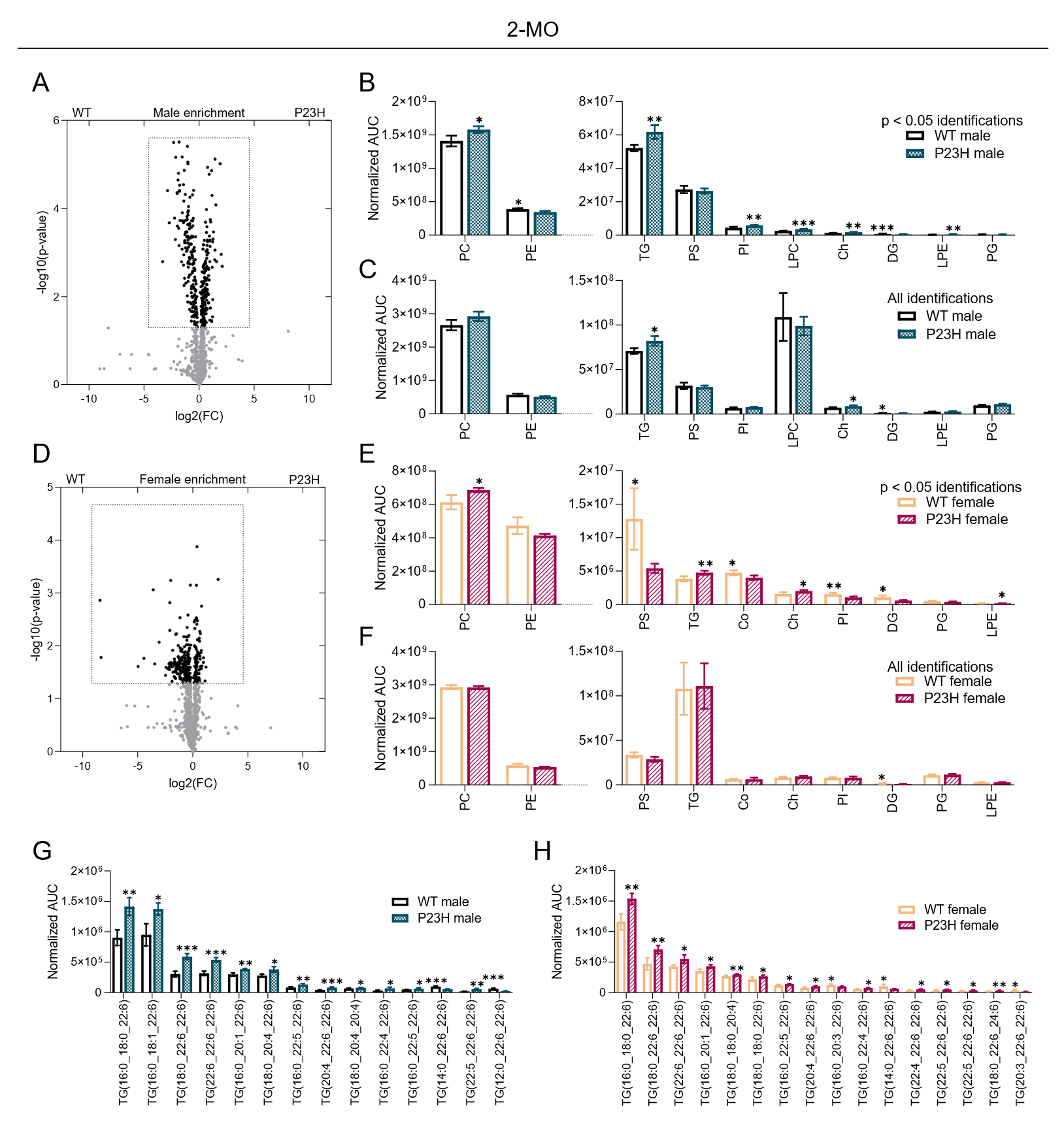
**

**Figure S15. Analysis of differentially expressed lipids between genotypes for both sexes at the age of 2 months.** A) Volcano plot of all identified lipids in WT and P23H males. Differentially expressed lipids (DELs) between WT males (n = 4) and P23H males (n = 3) are shown in black. B) Lipid group expression comparison between WT males and P23H males based on DELs only. C) Lipid group expression comparison between WT males and P23H males based on all lipid identifications. D) Volcano plot of all identified lipids in WT and P23H females. DELs between WT females (n = 4) and P23H females (n = 4) are shown in black. E) Lipid group expression comparison between WT females and P23H females based on DELs only. F) Lipid group expression comparison between WT females and P23H females based on all lipid identifications. G) Differentially expressed TGs with 20:4/22:5/22:6 carbon/double-bond sidechains between WT males and P23H males, and H) between WT females and P23H females. In all graphs, the asterisks indicate significant t-test results, * = p < 0.05, ** = p < 0.01 and *** = p < 0.001. The bar graph data is presented as mean ±SD. Co = coenzyme Q, Ch = Cholestrol, DG = diglyceride, LPC = lysophosphatidylcholine, LPE = lysophosphatidylethanolamine, PC = phosphatidylcholine, PE = phosphatidylethanolamine, PG = phosphatidylglycerol, PI = phosphatidylinositol, PS = phosphatidylserine, TG = triglyceride.

**Table S1. Protocol for scotopic electroretinography stimulation.** ISI, inter-stimulus interval time.

| **Step** | **Stimulus (cd•s/m^2^), green** | **ISI (s)** | **Repetitions** |
| --- | --- | --- | --- |
| 1 | 0.00001 | 1 | 25 |
| 2 | 0.00005 | 2 | 20 |
| 3 | 0.0001 | 2 | 20 |
| 4 | 0.0005 | 3 | 15 |
| 5 | 0.001 | 5 | 12 |
| 6 | 0.01 | 10 | 8 |
| 7 | 0.05 | 10 | 8 |
| 8 | 0.1 | 15 | 4 |
| 9 | 0.5 | 20 | 4 |
| 10 | 1 | 20 | 3 |
| 11 | 10 | 45 | 2 |
| 12 | 30 | 60 | 2 |

**Table S2. Protocol for photopic electroretinography stimulation.**

| **Step** | **Stimulus (****cd•s/m^2^), blue** | **ISI (s)** | **Repetitions** |
| --- | --- | --- | --- |
| 1 | 0.1 | 0.15 | 40 |
| 2 | 0.3 | 0.15 | 30 |
| 3 | 0.5 | 0.15 | 25 |
| 4 | 1 | 0.5 | 15 |
| 5 | 3 | 0.5 | 15 |
| 6 | 10 | 0.75 | 10 |
| 7 | 30 | 0.75 | 10 |
| 8 | 50 | 1 | 10 |

**Table S3. Primers for qPCR.**

| **Gene** | **Marker** | **Forward (Fw) – Reverse (Rv)** |
| --- | --- | --- |
| Estrogen receptor 1 (*Esr1*) | Sex hormone receptor | Fw: CCCGCCTTCTACAGGTCTAAT  Rv: CTTTCTCGTTACTGCTGGACAG |
| Estrogen receptor 2 (*Esr2*) | Sex hormone receptor | Fw: CTGTGCCTCTTCTCACAAGGA  Rv: TGCTCCAAGGGTAGGATGGAC |
| Androgen receptor (*Ar*) | Sex hormone receptor | Fw: CTGGGAAGGGTCTACCCAC  Rv: GGTGCTATGTTAGCGGCCTC |
| Vimentin (*Vim*) | Müller glia activation [2]. | Fw: TTCTCTGGCACGTCTTGACC  Rv: CTCCTGGAGGTTCTTGGCAG |
| Integrin subunit alpha M (*Itgam/Cd11b*) | Microglia marker activation [3]. | Fw: CTTTGGGAACCTCCGACCAG  Rv: CACCAAAGTGTCCAAGCCCA |
| Interleukin 6 (*Il6*) | Proinflammatory cytokine [4] | Fw: ATCCAGTTGCCTTCTTGGGACTGA  Rv: TAAGCCTCCGACTTGTGAAGTGGT |
| Tumour necrosis factor alpha (*Tnfa*) | Proinflammatory cytokine [5]. | Fw: TCTCATGCACCACCATCAAGGACT  Rv: ACCACTCTCCCTTTGCAGAACTCA |
| Interferon regulatory factor 8 (*Irf8*) | Reactive microglia [6]. | Fw: TTCAAGGCAGGTGGTGGT  Rv: GGATATGCCGCCTATGACAC |
| S100 calcium-binding protein B (*S100b*) | Gliosis [7]. | Fw: CTGGAGAAGGCCATGGTTGC  Rv: CTCCAGGAAGTGAGAGAGCT |
| Superoxide dismutase 1 (*Sod1*) | Oxidative stress, conversion of superoxide radicals into hydrogen peroxide [8]. | Fw: AACCAGTTGTGTTGTCAGGAC  Rv: CCACCATGTTTCTTAGAGTGAGG |
| Superoxide dismutase 2 (*Sod2*) | Oxidative stress, conversion of superoxide radicals into hydrogen peroxide [8] | Fw: CAGACCTGCCTTACGACTATGG  Rv: CTCGGTGGCGTTGAGATTGTT |
| Catalase (*Cat*) | Breakdown of hydrogen peroxide to water and oxygen [8] | Fw: GGAGGCGGGAACCCAATAG  Rv: GTGTGCCATCTCGTCAGTGAA |
| Glutathione peroxidase 4 (*Gpx4*) | Breakdown of hydrogen peroxide to water and oxygen, lipid peroxidation [8] | Fw: GATGGAGCCCATTCCTGAACC  Rv: CCCTGTACTTATCCAGGCAGA |
| Succinate Dehydrogenase Complex Flavoprotein Subunit A (*Sdha*) | Housekeeping gene 1 | Fw: GCTCCTGCCTCTGTGGTTGA  Rv: AGCAACACCGATGAGCCTG |
| Glyceraldehyde-3-Phosphate Dehydrogenase (*Gapdh*) | Housekeeping gene 2 | Fw: TGACGTGCCGCCTGGAGAAA  Rv: AGTGTAGCCCAAGATGCCCTTCAG |

**Table S4. Internal splash standards for phosphatidylcholine (PC), phosphatidylethanolamine (PE), phosphatidylserine (PS), phosphatidylglycerol (PG), phosphatidylinositol (PI), phosphatidic acid (PA), lysophosphatidylcholine (LPC), lysophosphatidylethanolamine (LPA), diglyceride (DG), triglyceride (TG), sphingomyelin (SM) and cholestryl ester (ChE) lipid groups.**

| Lipid group | Splash standard |
| --- | --- |
| PC | PC(33:1)+H+D7:(s) |
| PE | PE(15:0_18:1)+H+D7:(s) |
| PS | PS(33:1)+H+D7:(s) |
| PG | PG(15:0_18:1)+NH4+D7:(s) |
| PI | PI(18:1_15:0)+NH4+D7:(s) |
| PA | PA(15:0_18:1)+NH4+D7:(s) |
| LPC | LPC(18:1)+H+D7:(s) |
| LPE | LPE(18:1)+H+D7:(s) |
| DG | DG(33:1)+H+D7:(s) |
| TG | TG(15:0_15:0_18:1)+NH4+D7:(s) |
| SM | PG(15:0_18:1)+NH4+D7:(s) |
| ChE | Ch-D7+H-H2O |

**Supplemental Data 1 (separate file).** Raw data and animal information file.

**Supplemental Data 2 (separate file).** Lipidomics data file.

**Supplemental Data 3 (separate file).** Re-analysis of publicly available human bulk retina RNA-seq dataset.

**References**

1. H. Leinonen, J. Zhang, L. Occelli, et al., A combination treatment based on drug repurposing demonstrates mutation-agnostic efficacy in pre-clinical retinopathy models. *Nat. Commun*. **15**, 5943 (2024).
2. A. Bringmann, T. Pannicke, J. Grosche, et al., Müller cells in the healthy and diseased retina. *Prog. Retin. Eye Res.* **25**, 397-424 (2006), https://doi.org/10.1016/j.preteyeres.2006.05.003.
3. R. Zauhar, J. Biber, Y. Jabri, et al., As in Real Estate, Location Matters: Cellular Expression of Complement Varies Between Macular and Peripheral Regions of the Retina and Supporting Tissues. *Front. Immunol.* **13**, 895519 (2022), https://doi.org/10.3389/fimmu.2022.895519.
4. T. Tanaka, M. Narazaki, T. Kishimoto, IL-6 in Inflammation, Immunity, and Disease. *Cold Spring Harb. Perspect. Biol.* **6**, a016295 (2014), https://doi.org/10.1101/cshperspect.a016295.
5. M. McDermott, TNF and TNFR biology in health and disease. *Cell Mol. Biol.* **47**, 619-635 (2001).
6. P. Zhang, A. Schlecht, J. Wolf, et al., The role of interferon regulatory factor 8 for retinal tissue homeostasis and development of choroidal neovascularisation. *J. Neuroinflammation.* **18**, 215 (2021), https://doi.org/10.1186/s12974-021-02230-y
7. F. Michetti, N. D’Ambrosi, A. Toesca, et al., The S100B story: from biomarker to active factor in neural injury. *J. Neurochem.* **148**, 168-187 (2019), https://doi.org/10.1111/jnc.14574.
8. M. Azam, B. Jastrzebska, Mechanisms of Rhodopsin-Related Inherited Retinal Degeneration and Pharmacological Treatment Strategies*. Cells.* **14**, 49 (2025), https://doi.org/10.3390/cells14010049.
